# Antiphospholipid Antibodies Increase the Risk of Fetal Growth Restriction: A Systematic Meta-Analysis

**DOI:** 10.1155/2022/4308470

**Published:** 2022-01-31

**Authors:** Jinfeng Xu, Daijuan Chen, Yuan Tian, Xiaodong Wang, Bing Peng

**Affiliations:** ^1^Department of Obstetrics and Gynecology, West China Second University Hospital of Sichuan University, Chengdu, China; ^2^West China School of Medicine, Sichuan University, Chengdu 610041, Sichuan, China; ^3^The Key Laboratory of Birth Defects and Related Diseases of Women and Children, Sichuan University, Ministry of Education, Chengdu 610041, China

## Abstract

**Objective:**

Antiphospholipid syndrome (APS) is a chronic autoimmune disease with a high prevalence in females. Published data have identified pregnant women with APS may suffer from recurrent miscarriage, fetal death. However, the association between antiphospholipid antibody (aPL) and fetal growth restriction (FGR) remains controversial. This study aims to systematically review the literature on population-based studies investigating an association between aPL and FGR.

**Methods:**

The literature was searched on 1 November, 2021, using Ovid MEDLINE, Embase, and Cochrane Central Register of Controlled Trials (CENTRAL), following the MOOSE checklist. Study inclusion criteria focused on peer-reviewed published articles that reported an association between aPL and FGR. Quality assessment was performed based on the Newcastle-Ottawa scale. The between-study heterogeneity was assessed by the Q test. Publication bias was assessed by funnel plots.

**Results:**

Twenty-two studies (with 11745 pregnant women) were included in the final analysis. Pooled odds ratio for association of aPL, anticardiolipin antibodies (ACA), anti-beta2 glycoprotein 1 antibodies (*β*2GP1), and FGR was 1.26 (95%CI 1.12, 1.40), 2.25 (95%CI 1.55, 2.94), and 1.31 (95% CI 1.12, 1.49), respectively. Lupus anticoagulant (LA) did not increase the chance of FGR (OR 0.82, 95%CI 0.54, 1.10).

**Conclusions:**

Our meta-analysis showed that aPL increased the risk of FGR. The risk of FGR varies with the aPL types. ACA and *β*2GP1 are strongly associated with FGR. There are currently insufficient data to support a significant relationship between LA and FGR.

## 1. Introduction

Antiphospholipid syndrome (APS) is an autoimmune condition, which may potentially cause adverse pregnancy outcomes. The current diagnosis of APS requires at least one laboratory and clinical criterion each [1]. Laboratory criteria require the presence of one of the antiphospholipid antibodies (aPL) detected in patient's serum or plasma on two separate occasions ≥12 weeks apart, mainly including anticardiolipin antibodies (ACA), lupus anticoagulant (LA), and anti-beta2 glycoprotein 1 antibodies (*β*2GP1) [1]. One of the clinical criteria includes the occurrence of one or more premature births of a morphologically normal neonate before the 34th week of gestation because of placental insufficiency. One of the features of placental insufficiency is fetal growth restriction (FGR), defined as a fetus that has not achieved his or her growth potential [2]. To assess for fetal growth restriction, four biometric measures are commonly used, generating an estimated fetal weight. Some guidelines define FGR as ultrasound estimated fetal weight of less than 10th percentile for gestational age on the reference chart [2]. FGR is the result of a variety of different maternal, fetal, and placental conditions, resulting in severe perinatal mortality and morbidity [3]. Furthermore, FGR children are at increased risk of minor cognitive deficits, poor school performance, and metabolic syndrome in adulthood [4]. APS is one of the common etiologies of FGR [2]. The incidence of FGR in APS patients is reported to be between 6.7% and 16.0% [5–8]. Children born to mothers suffering from APS are associated to FGR and low birth weights, even undergoing prophylactic treatment with aspirin and low-dose non-fractioned heparin [9]. However, the association between aPL and FGR still remains obscure in spite of the great number of studies having focused on the issue. Therefore, we conducted a systematic review and meta-analysis to investigate an association between aPL and FGR.

## 2. Materials and Methods

### 2.1. Information Sources and Search Strategy

We followed the methods of Xu et al. [10]. A systematic search of the MEDLINE, EMBASE, and Cochrane Central Register of Controlled Trials (CENTRAL) database was conducted up to 1 November, 2021. Combinations of the following keywords and MESH search terms were used: “fetal growth restriction,” “fetal growth retardation,” “intrauterine growth retardation,” “small for gestational age,” “antiphospholipid antibody,” “anticardiolipin antibody,” “lupus anticoagulant,” and “beta2-glycoprotein I.” The search was limited to human studies published in English. Unpublished studies were not included. References of included studies and reviews were also hand searched for potential studies. A detailed description of the search strategy can be found in the Supplementary material (Appendix S1).

### 2.2. Study Selection

Two reviewers (JFX and YT) independently evaluated the titles and abstracts. Duplications were removed using EndNote online software. Disagreements were resolved by the third reviewer (BP). Only case-control study, cohort study, and cross-sectional study were evaluated. We excluded the studies with the following criteria: (1) presence of other autoimmune diseases; (2) patients undergoing serious internal and surgical diseases; and (3) multiple pregnancies.

### 2.3. Data Extraction

We used a standard form to extract data, including the journal, first author's name, publication year, country, study design, time of study conduct, the age of patients, ascertainment method of diseases and control or comparison group selection, types of aPL, the definition of FGR, risk factors for FGR, number of population analyzed, odd ratios (ORs) or relative risks (RRs) and 95% confidence intervals (95%CIs), and statistical methods. When effect estimates for association between aPL and FGR were not listed in the original article but enough information was available, their effect estimates were calculated by STATA 12.0 statistical software. Authors were contacted if important information was lost. Data extraction was conducted independently by two of the reviewers (JFX and YT).

### 2.4. Primary Outcomes

The outcome indicator was the occurrence of FGR. The FGR positivity is defined as ultrasound estimated fetal weight less than 10th percentile [7] or birthweight <5th percentile [11] or birthweight < -2SD unit [12].

#### 2.4.1. Risk of Bias, Summary Measures, and Synthesis of the Results

Two researchers (JFX and DJC) independently assessed the methodological quality of studies, using the Newcastle-Ottawa quality assessment scale (NOS) for cohort and case-control study [13]. A score of 6–9 suggested a high level of quality and low risk of bias.

We used the MOOSE checklist and PRISMA guidelines for this systematic review [14, 15]. We performed meta-analysis to calculate odd ratios (ORs) and 95% confidence intervals (CIs) using the Mantel–Haenszel method with STATA 12.0 statistical software. Heterogeneity was measured using the *I*^2^ statistic. A random effects model was used because we assumed the presence of heterogeneity in these clinical studies with different clinical settings [16]. *P* values <0.05 were considered statistically significant. We conducted a sensitivity analysis to exclude these studies, which varied dramatically from all other included studies in methodology or findings. Subgroup analysis by study type was also conducted to assess the potential sources of heterogeneity. Publication bias was assessed by funnel plots and Begg's test.

## 3. Results

### 3.1. Study Selection and Study Characteristics

**Figure 1:**
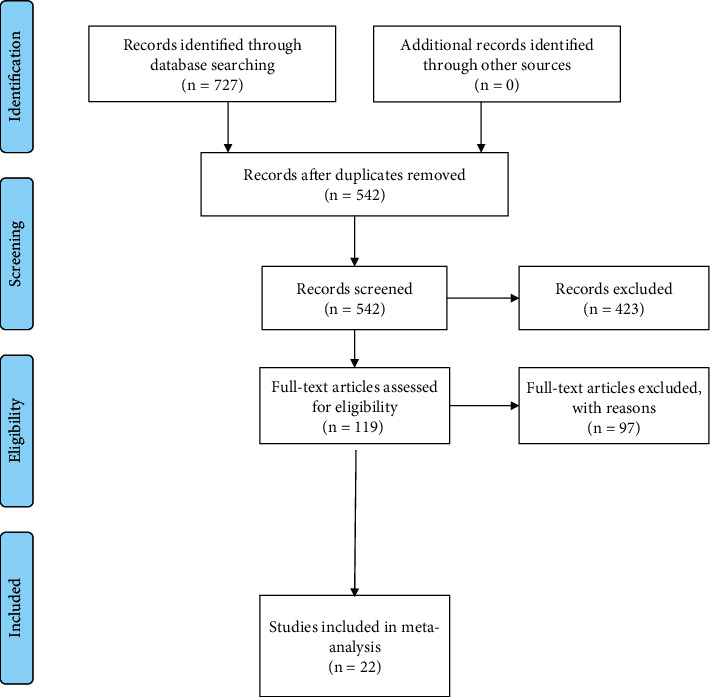
Study flow diagram.

727 references were identified through electronic searches of MEDLINE (*n* = 358), Embase (*n* = 265), and Cochrane Central Register of Controlled Trials (CENTRAL) database (*n* = 104). After removing 173 duplicates using EndNote software (EndNote X7) and 12 duplicates manually, the titles and abstracts of 542 papers were scrutinized. The reference lists of relevant reviews were hand searched. A total of 119 articles were assessed for eligibility. Of them, 97 were excluded for the reasons, such as presence of other autoimmune diseases (*n* = 56), patients undergoing serious internal and surgical diseases (*n* = 39), and multiple pregnancies (*n* = 2). Of the 119 citations identified, 22 articles with 11745 cases (Table 1) were selected for detailed assessment [6, 7, 11, 12, 17–27, 29–34]. Details of the study selection process are shown in the PRISMA flow diagram (Figure 1). Furthermore, we evaluated risk factors for FGR of studies including hypertension, pre-eclampsia, eclampsia, diabetes mellitus, Iron deficiency anemia, renal involvement, smoking, hyperthyroidism, obesity, dyslipidaemia, and thrombosis (Table 1).

Detailed description of key characteristics for the 22 included manuscripts is shown in Table 1. With regard to study design, 16 (72.7%; 8224 cases) were cohort and 6 (27.3%; 3521 cases) were case-control studies. Sample size varied from 38 to 1,616 women. The age of patients ranged from 25 to 41.5 years. The definition of FGR from eighteen studies was ultrasound estimated fetal weight less than 10th percentile for gestational age [7], and the definition of three studies was birthweight <5th percentile [11]; one study was birthweight < -2SD [12]. The quality score of the included studies can be found in the Supplementary material (Table S1). The lowest quality score of NOS was 5, and the highest was 8. Eight studies received eight stars, three studies received seven stars, five studies received six stars, and six studies received five stars.

### 3.2. Risk of Bias within Studies

Bias assessment within studies is shown in Table S1.

### 3.3. Synthesis of the Results

#### 3.3.1. Antiphospholipid Antibodies

**Figure 2:**
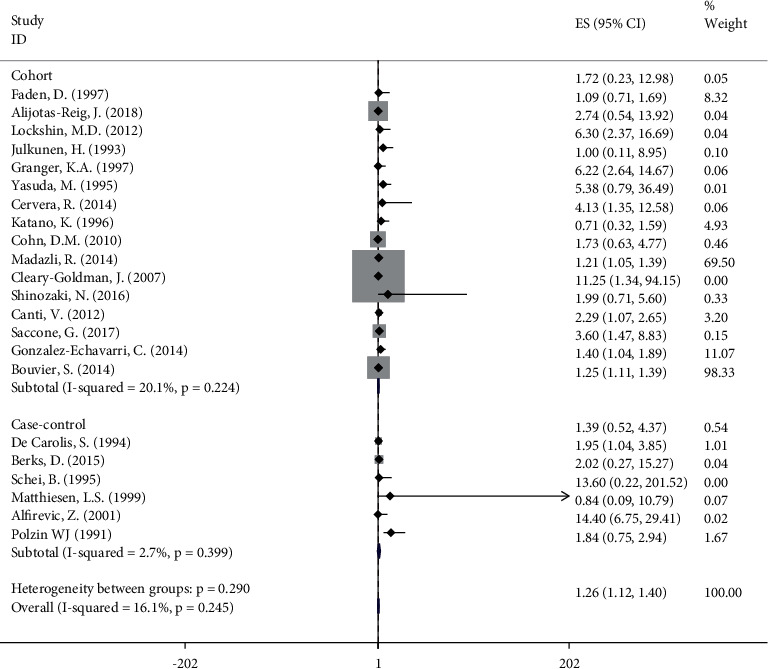
Meta‐analysis of all studies of participants with antiphospholipid antibody positivity. OR, odds ratio; 95% CI, 95% confidence interval.

In 10 out of the twenty-two selected studies, statistically significant association can be seen between aPL and FGR (OR 1.26, 95%CI 1.12, 1.40) [6, 12, 21, 22, 24, 25, 27–29, 31]. Nine studies found a positive but not statistically significant relationship [7, 11, 17, 18, 26, 30, 32, 33]. Furthermore, three studies showed a non-significant association between aPL and FGR [19, 23, 34]. According to subgroup analysis of study type, aPL revealed a statistically significant association with FGR in the group of cohort studies (OR 1.25, 95%CI 1.11, 1.39). However, in the subgroup of case-control studies, aPL did not reveal a statistically significant association with FGR (OR 1.84, 95%CI 0.75, 2.94). Low heterogeneity (*I*^2^ = 16.1%, *P* = 0.245) was observed in our analysis (Figure 2).

### 3.4. Anticardiolipin Antibody

**Figure 3:**
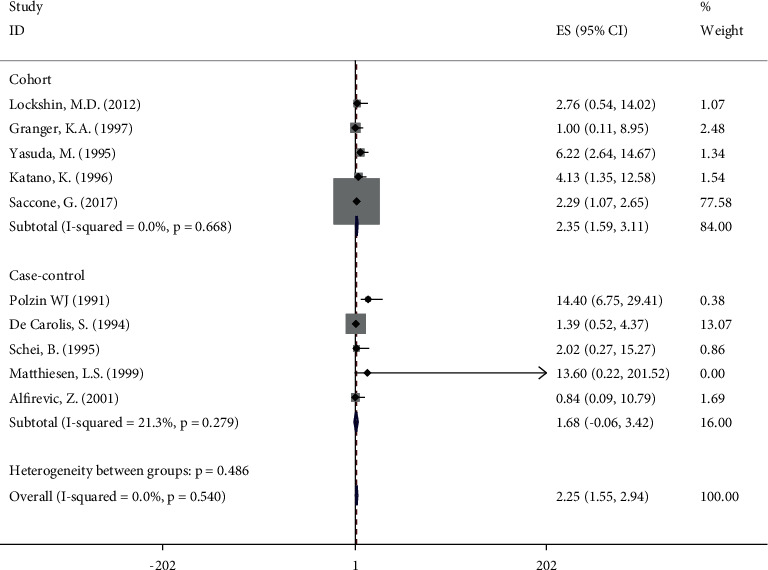
Meta‐analysis of all studies of participants with anticardiolipin antibody positivity. OR, odds ratio; 95% CI, 95% confidence interval.

Results derived from ten included studies [6, 18, 19, 21, 22, 29, 30, 32–34] with 4976 cases suggested a statistically significant association for ACA and FGR (OR 2.25, 95%CI 1.55, 2.94) (Figure 3). Similarly, we can find the relationship between ACA and FGR among cohort studies (OR 2.35, 95%CI 1.59, 3.11). However, among the group of case-control studies, ACA did not reveal a statistically significant association with FGR (OR 1.68, 95%CI −0.06, 3.42). No heterogeneity (*I*^2^ = 0%, *P* = 0.540) was observed in analysis of ACA.

### 3.5. Anti-Beta 2 Glycoprotein 1 Antibody

**Figure 4:**
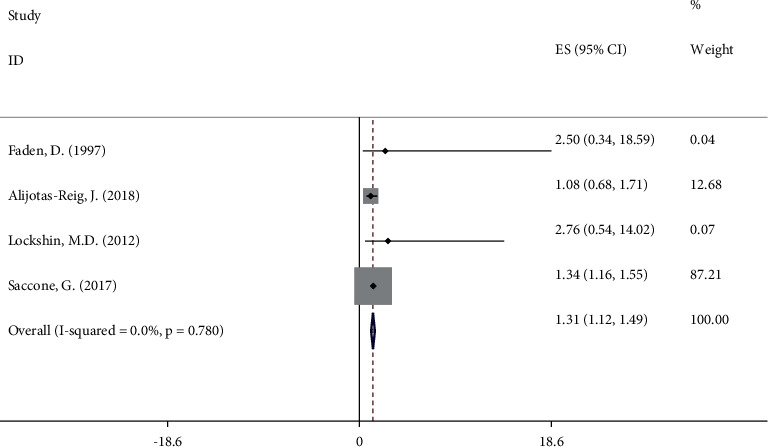
Meta‐analysis of all studies of participants with anti-beta2 glycoprotein 1 antibody positivity. OR, odds ratio; 95% CI, 95% confidence interval.

Figure 4 shows that there was a strong association between *β*2GP1 and FGR (OR 1.31; 95%CI 1.12, 1.49) among four cohort studies [7, 17, 18, 22] with 2562 cases. No heterogeneity (*I*^2^ = 0%, *P* = 0.780) was observed in analysis of *β*2GP1.

### 3.6. Lupus Anticoagulant

**Figure 5:**
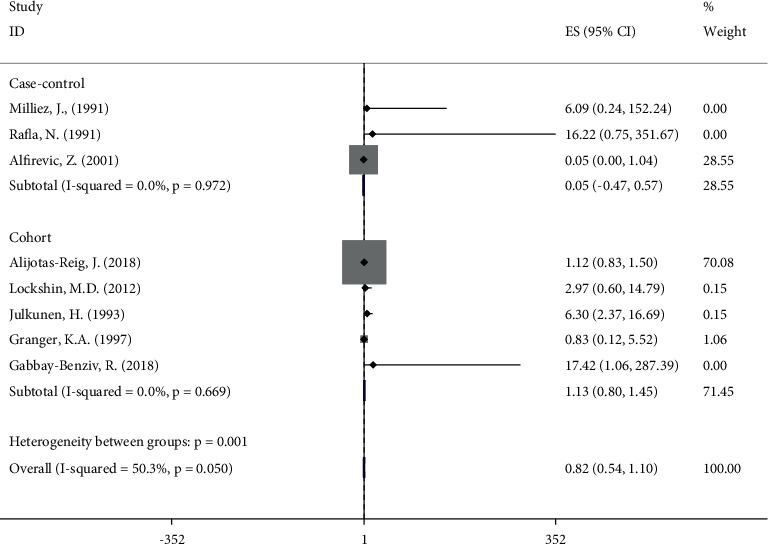
Meta‐analysis of all studies of participants with lupus anticoagulant positivity. OR, odds ratio; 95% CI, 95% confidence interval.

Figure 5 presents the OR and 95%CI for studies about LA. All studies for inclusion [7, 12, 18, 19, 29, 34–36] demonstrated that LA did not increase the chance of FGR (OR 0.82, 95%CI 0.54, 1.10), only two studies [12, 35] reported statistically significant associations, and six studies [7, 18, 19, 29, 34, 36] did not report statistically significant associations. The heterogeneity among studies was slightly significant (*I*^2^ = 50.3%, *P* = 0.05).

### 3.7. Publication Bias

Funnel plots were used to explore the publication bias of the included studies (Figures S1A–S1C). Funnel plot for aPL, ACA, and LA showed minor asymmetry, and Begg's test (Figures S2A–S2D) was not significant with *P* = 1.000, *P* = 0.721, *P* = 0.734, and *P* = 1.000, respectively.

### 3.8. Sensitivity Analysis

A sensitivity analysis for each meta-analysis with at least three studies was conducted. Within each meta-analysis, each study was taken out, respectively, to assess for its influence on the overall risk estimates.

## 4. Discussion

The association of FGR with APS has been reported by some studies [6, 37–39]. One case-control study [21] demonstrated that aPL positivity increased the risk of FGR by approximately twofold compared to controls. Similarly, Spegiorin et al. reported that newborns born to mothers suffering from APS were associated with FGR and low birth weight [9]. The rate of FGR varied between 16.1% and 51.3% [40–44]. The morbidity and mortality associated with FGR and fetal death represent a significant disease burden for women and their children. However, few studies have examined the association between aPL and FGR in a systematic manner.

In this systematic review and meta-analysis, we aimed to provide a comprehensive overview of available evidence on the association between aPL and FGR. The aPL refers to three tests including ACA, LA, and *β*2GP1. In the present study, we demonstrated that women with aPL positivity have a higher risk of FGR. According to our analysis, the presence of aPL increases the odds of FGR 1.26 times (95%CI 1.12, 1.40). This is concordant with recently published data by Högdén et al. showing that the persistent presence of aPL is associated with FGR [20, 37]. Apart from thrombosis, the complement system seems to mediate aPL-related FGR [45]. Some reported that mice infused with aPL had exhibited FGR with a 45% decrease in fetal weight [46], while mice with complement deficiency showed protection against aPL-induced pregnancy complications. aPL positivity was associated with higher rates of FGR [22], emphasizing the need for a multispecialty approach in the care of these patients with respect to close monitoring and early recognition of clinical signs of FGR [47].

Screening for ACA is most commonly used in clinical practice as one of the criteria for classification of APS. Some studies reported that the incidence of FGR was higher in positive ACA than in negative ACA cases [38, 48]. The positive predictive values for FGR of a positive result were 38.0% for IgG ACA [38]. Our study confirms these findings. According to our analysis, ACA is the strongest risk factor for FGR among the aPL (OR 2.25, 95%CI 1.55, 2.94). The cause of FGR might be related to vascular constriction and thromboses of the placenta. ACA inhibited the production of prostacyclin, which is a potent vasodilator and an inhibitor of platelet aggregation [49]. Other mechanisms for ACA-mediated damage may also attribute to obstetric complications, such as decreased production of IL-3 in maternal plasma, possible interference of aPL with signal transduction in trophoblast cells, and prevention of hormone production by the placenta [50].

*β*2GP1 are rarely the sole antibodies detected in patients with clinical features of APS. However, it is a main target of antiphospholipid antibodies, playing a role in pathogenesis of adverse obstetric outcomes [1]. Saccone et al. suggested that *β*2GP1 are the ones associated with the lowest live birth rate and highest incidences of FGR, very preterm FGR, and stillbirth compared with ACA or with LA alone [22]. We found, in agreement with previous reports, a strong association between *β*2GP1 and FGR (OR 1.31; 95% CI 1.12, 1.49) among four cohort studies [7, 17, 18, 22]. We believe that early screening for *β*2GP1 may be beneficial to APS patients who have a history of FGR.

Data from a case-control study of 2257 pregnancies reported that significantly more women positive for LA had pregnancies complicated by FGR compared with women who were LA negative [51]. Furthermore, a meta-analysis concluded that LA positivity increases the odds of FGR 4.65 times (95%CI 1.29–16.71) [52]. Despite pooling data from 8 studies, lack of adequate power has remained a serious limiting factor in our ability to draw a firm conclusion on the association between LA and FGR (OR 0.82, 95%CI 0.54, 1.10). Furthermore, although subgroup analysis had been conducted, both cohort and case-control groups supported a surprising lack of correlation between LA and FGR. In contrast, based on a prospective multicenter observational study, LA positivity was reported as a predictor of adverse pregnancy outcomes regardless of the association with ACA or *β*2GP1 positivity [18]. Therefore, further large multicenter studies are warranted to investigate the association.

### 4.1. Management of aPL Positivity during Pregnancy

Currently, the treatment strategies to prevent APS-related obstetric complications include antiplatelet (low-dose aspirin) [53], anticoagulan (unfractionated heparin; low molecular weight heparin) [53], and/or immunomodulatory therapies (hydroxychloroquine and corticosteroids) [54, 55]. For refractory obstetric APS, more and more evidence indicates the use of intravenous immunoglobulins [56] or plasmapheresis [57].

### 4.2. Strengths and Limitations

Our study is the most comprehensive review evaluating the association between aPL and FGR by the type of aPL. Also, we conducted separate meta-analyses by the types of aPL. Furthermore, we evaluated risk factors for FGR of studies included. Finally, half of the included studies were published after 2010, suggesting a minor effect of publication years. Furthermore, we evaluated risk factors for FGR of studies included. However, our study also has some limitations. Firstly, some included studies had limited number of FGR cases, which might generate spurious associations. Additionally, most patients with FGR were treated with drugs like aspirin and heparin, which might lead to the underestimation of risk. Finally, different definitions of FGR can cause more variations. Therefore, caution is needed when interpreting the results.

Recently, a systematic review and meta-analysis evaluated pregnancy outcomes in patients with APS [58]. However, their search was up to 2016 and had a small number of studies with eight studies included. Furthermore, although the total number of participants with APS was large, the large population size of the only one study instantly might lead to a higher heterogeneity in the meta-analysis [59].

## 5. Conclusions

Our meta-analysis showed that aPL positivity increased the risk of FGR. Furthermore, the risk of FGR varies with the aPL antibody type. Therefore, it is necessary to measure aPL when FGR occurs. Women who have experienced adverse pregnancy outcomes, particularly severe FGR, should be screened for the presence of aPL. In order to generate the best evidence, we recommend future population-based studies to further investigate the association between aPL and FGR by adopting a prospective cohort study approach with large study populations, carefully selected disease-free controls, good adjustment for potential confounding factors, and longer duration of follow-up through multiple pregnancies.

## Figures and Tables

**Table 1:** Characteristics of included studies.

Year	Author	Country	Study design	Study period	Study Size	Age (years)	Types of aPL	NOS score	Risk factors for FGR (number)	Definition of FGR
1997	Faden et al. [17]	Italy	Cohort	1993–1994	510	29.3 ± 3.4 (21–40)	*β*2GP1	7	NR	Birthweight < 5th percentile
2018	Alijotas-Reig et al. [7]	European	Cohort	2010–2018	1000	35.2 ± 5.9	LA *β*2GP1	8	Smoking 152, obesity 86, dyslipidaemia 22, DM 18	Birthweight ≤ 10th percentile
2012	Lockshin et al. [18]	USA	Cohort	2003–2011	302	32 ± 2.5	ACA *β*2GP1 LA	6	Thrombosis 25	Birthweight ≤ 10th percentile
1993	Julkunen et al. [12]	Finland	Cohort	1987–1991	536	41.5 (23–39)	LA	5	NR	Birthweight ≤ 2SD unit
1997	Granger and Farquharson [19]	Liverpool	Cohort	1992–1994	387	31 ± 1.3	ACA LA	5	NR	Birthweight ≤ 10th percentile
1995	Yasuda et al. [6]	Japan	Cohort	1991–1992	860	29.8 ± 4.3	ACA	8	Hyperthyroidism 8	Birthweight ≤ 10th percentile
2014	Cervera et al. [20]	European	Cohort	1999–2009	188	38 ± 3.7	aPL	5	Hypertension 3	Birthweight ≤ 10th percentile
1996	Katano et al. [21]	Japan	Cohort	1990–1992	1125	32.5 ± 4.1	ACA	8	NR	Birthweight ≤ 10th percentile
2017	Saccone et al. [22]	Italy	Cohort	2007–2016	750	28.4 ± 7.7	ACA *β*2GP1 LA	8	Smoking 59, DM 29	Birthweight ≤ 10th percentile
2010	Cohn et al. [23]	UK	Cohort	1986–2006	693	32 ± 5.6	aPL	8	NR	Birthweight ≤ 10th percentile
2014	Madazli et al. [11]	Turkey	Cohort	2002–2011	65	28.8 ± 4.3	aPL	5	Renal involvement 9	Birthweight < 5th percentile
2007	Cleary-Goldman et al. [24]	USA	Cohort	2005–2007	151	NR	aPL	5	NR	Birthweight < 5th percentile
2016	Shinozaki et al. [25]	Japan	Cohort	2009–2014	38	32 (29–40)	aPL	7	NR	Birthweight ≤ 10th percentile
2012	Canti et al. [26]	Italy	Cohort	2001–2009	156	34.0 ± 4.5	aPL	6	NR	Birthweight ≤10th percentile
2014	Bouvier et al. [27]	France	Cohort	1970–2010	1313	30 (16–44)	aPL	8	PE 89	Birthweight ≤ 10th percentile
2014	Gonzalez-Echavarri et al. [28]	Spain	Cohort	2011–2013	150	NR	aPL	6	NR	Birthweight ≤ 10th percentile
1991	Polzin et al. [29]	USA	Case control	1990–1991	1616	NR	ACA	8	IDA 3, smoking 2	Birthweight ≤10th percentile
1994	De Carolis et al. [30]	Italy	Case control	1986–1992	365	NR	ACA	6	NR	Birthweight ≤ 10th percentile
2015	Berks et al. [31]	Netherlands	Case control	1985–2010	844	29 (17–43)	aPL	8	Eclampsia 28	Birthweight ≤ 10th percentile
1995	Schei et al. [32]	Norway	Case control	NR	414	25 (17–43)	ACA	8	Smoking 3	Birthweight ≤ 10th percentile
1999	Matthiesen et al. [33]	Sweden	Case control	1991–1992	213	28 (19–34)	ACA	5	NR	birthweight ≤ 10th percentile
2001	Alfirevic et al. [34]	UK	Case control	1997–1998	69	29 (15–42)	ACA LA	6	Smoking 7	Birthweight ≤ 10th percentile

## Data Availability

The data that support the findings of this study are included in this article and available from the corresponding author upon reasonable request.

## Supplementary Materials

Supplementary Materials Appendix S1: search strategy. Table S1: the Newcastle-Ottawa quality assessment scale score of each included study. Figure S1A: funnel plot of all studies positive for antiphospholipid antibodies. Figure S1B: funnel plot of all studies positive for anticardiolipin antibodies. Figure S1C: funnel plot of all studies positive for lupus anticoagulant. Figure S2A: Begg's test of antiphospholipid antibodies. Figure S2B: Begg's test of anticardiolipin antibodies. Figure S2C: Begg's test of anti-beta2 glycoprotein 1 antibodies. Figure S2D: Begg's test of lupus anticoagulant.
