# Supplementary material for: Antiphospholipid Antibodies Increase the Risk of Fetal Growth Restriction: A Systematic Meta-Analysis
Source: Int J Clin Pract. 2022 Jan 31;2022:4308470. doi: 10.1155/2022/4308470 (PMC9159204; doi:10.1155/2022/4308470)
Supplement: Supplementary Materials — Appendix S1: search strategy. Table S1: the Newcastle-Ottawa quality assessment scale score of each included study. Figure S1A: funnel plot of all studies positive for antiphospholipid antibodies. Figure S1B: funnel plot of all studies positive for anticardiolipin antibodies. Figure S1C: funnel plot of all studies positive for lupus anticoagulant. Figure S2A: Begg's test of antiphospholipid antibodies. Figure S2B: Begg's test of anticardiolipin antibodies. Figure S2C: Begg's test of anti-beta2 glycoprotein 1 antibodies. Figure S2D: Begg's test of lupus anticoagulant. [file 4308470.f1.zip › 4308470.f1/Figure S2A .pdf]

Figure S2A **The Begg's test of antiphospholipid antibodies.**

```
Begg's   Test

adj. Kendall's Score (P-Q) =     -1
     Std. Dev. of Score    =     35.46
     Number of Studies     =     22
     z     =    -0.03
     Pr > z    =    0.978
     z     =    0.00(continuity     corrected)
     Pr > z    =    1.000     (continuity     corrected)
```
