# Supplementary material for: Antiphospholipid Antibodies Increase the Risk of Fetal Growth Restriction: A Systematic Meta-Analysis
Source: Int J Clin Pract. 2022 Jan 31;2022:4308470. doi: 10.1155/2022/4308470 (PMC9159204; doi:10.1155/2022/4308470)
Supplement: Supplementary Materials — Appendix S1: search strategy. Table S1: the Newcastle-Ottawa quality assessment scale score of each included study. Figure S1A: funnel plot of all studies positive for antiphospholipid antibodies. Figure S1B: funnel plot of all studies positive for anticardiolipin antibodies. Figure S1C: funnel plot of all studies positive for lupus anticoagulant. Figure S2A: Begg's test of antiphospholipid antibodies. Figure S2B: Begg's test of anticardiolipin antibodies. Figure S2C: Begg's test of anti-beta2 glycoprotein 1 antibodies. Figure S2D: Begg's test of lupus anticoagulant. [file 4308470.f1.zip › 4308470.f1/Appendix S1.doc]

**Appendix S1. Search Strategy**

1. Antiphospholipid Antibod*.mp.

2. Antibod*, Antiphospholipid.mp.

3. Anticardiolipin Antibod*.mp.

4. Antibod*, Anticardiolipin.mp.

5. Syndrome, Antiphospholipid.mp.

6. Hughes Syndrome.mp.

7. Syndrome, Hughes.mp.

8. Antiphospholipid Antibody Syndrome.mp.

9. Antibody Syndrome, Antiphospholipid.mp.

10. Antiphospholipid Antibody Syndromes.mp.

11. Syndrome, Antiphospholipid Antibody.mp.

12. Anti-Phospholipid Antibody Syndrome.mp.

13. Anti Phospholipid Antibody Syndrome.mp.

14. Antibody Syndrome, Anti-Phospholipid.mp.

15. Syndrome, Anti-Phospholipid Antibody.mp.

16. Anti-Phospholipid Syndrome.mp.

17. Anti Phospholipid Syndrome.mp.

18. Syndrome, Anti-Phospholipid.mp.

19. beta 2 Glycoprotein I.mp.

20. Apo H.mp.

21. Endothelial Cell-Viability Maintaining Factor.mp.

22. Endothelial Cell Viability Maintaining Factor.mp.

23. beta2-Glycoprotein I.mp.

24. beta2 Glycoprotein I.mp.

25. EC-VMFa.mp.

26. EC VMFa.mp.

27. Anticardiolipin Cofactor.mp.

28. Cofactor, Anticardiolipin.mp.

29. Apolipoprotein H.mp.

30. Inhibitor, Lupus Coagulation.mp.

31. Coagulation Inhibitor, Lupus.mp.

32. Lupus Anticoagulant.mp.

33. Anticoagulant, Lupus.mp.

34. Fetal Growth Restriction.mp.

35. Fetal Growth Retardation.mp.

36. Growth Retardation, Intrauterine.mp.

37. Intrauterine Growth Restriction.mp.

38. Small for gestational age

39. 1 or 2 or 3 or 4 or 5 or 6 or 7 or 8 or 9 or 10 or 11 or 12 or 13 or 14 or 15 or 16 or 17 or 18 or 19 or 20 or 21 or 22 or 23 or 24 or 25 or 26 or 27 or 28 or 29 or 30 or 31 or 32 or 33

40. 34 or 35 or 36 or 37 or 38

41. 39 and 40

42. limit 41 to english language

43. limit 42 to human

44. limit 43 to yr="1883 -2021"

45. remove duplicates from 44
