# Supplementary material for: Antiphospholipid Antibodies Increase the Risk of Fetal Growth Restriction: A Systematic Meta-Analysis
Source: Int J Clin Pract. 2022 Jan 31;2022:4308470. doi: 10.1155/2022/4308470 (PMC9159204; doi:10.1155/2022/4308470)
Supplement: Supplementary Materials — Appendix S1: search strategy. Table S1: the Newcastle-Ottawa quality assessment scale score of each included study. Figure S1A: funnel plot of all studies positive for antiphospholipid antibodies. Figure S1B: funnel plot of all studies positive for anticardiolipin antibodies. Figure S1C: funnel plot of all studies positive for lupus anticoagulant. Figure S2A: Begg's test of antiphospholipid antibodies. Figure S2B: Begg's test of anticardiolipin antibodies. Figure S2C: Begg's test of anti-beta2 glycoprotein 1 antibodies. Figure S2D: Begg's test of lupus anticoagulant. [file 4308470.f1.zip › 4308470.f1/Table S1.doc]

**Table S1. The Newcastle–Ottawa quality assessment Scale score of each included study.**

| **Study** | **Selection** | **Comparability** | **Outcome** | **Total score** |
| --- | --- | --- | --- | --- |
| Polzin WJ 1991 | ＊＊＊＊ | ＊ | ＊＊＊ | 8 |
| Faden, D. 1997 | ＊＊＊＊ | ＊ | ＊＊ | 7 |
| Alijotas-Reig, J. 2019 | ＊＊＊＊ | ＊＊ | ＊＊ | 8 |
| Lockshin, M.D. 2012 | ＊＊ | ＊＊ | ＊＊ | 6 |
| Julkunen, H. 1993 | ＊＊ | ＊ | ＊＊ | 5 |
| Granger, K.A. 1997 | ＊＊＊ | ＊ | ＊ | 5 |
| De Carolis, S. 1994 | ＊＊ | ＊ | ＊＊ | 5 |
| Yasuda, M. 1995 | ＊＊＊＊ | ＊＊ | ＊＊ | 8 |
| Cervera, R. 2015 | ＊＊ | ＊ | ＊＊ | 5 |
| Katano, K. 1996 | ＊＊＊＊ | ＊＊ | ＊＊ | 8 |
| Saccone, G. 2017 | ＊＊＊ | ＊＊ | ＊＊＊ | 8 |
| Cohn, D.M. 2010 | ＊＊＊ | ＊＊ | ＊＊＊ | 8 |
| Madazli, R. 2014 | ＊＊ | ＊ | ＊＊ | 5 |
| Cleary-Goldman,J. 2007 | ＊＊ | ＊＊ | ＊ | 5 |
| Berks, D. 2015 | ＊＊＊＊ | ＊＊ | ＊ | 7 |
| Shinozaki, N. 2016 | ＊＊＊ | ＊＊ | ＊＊ | 7 |
| Canti, V. 2012 | ＊＊＊ | ＊ | ＊＊ | 6 |
| Schei, B. 1995 | ＊＊＊ | ＊＊ | ＊＊＊ | 8 |
| Matthiesen, L.S. 1999 | ＊＊ | ＊＊ | ＊ | 5 |
| Alfirevic, Z. 2001 | ＊＊＊ | ＊＊ | ＊ | 6 |
| Bouvier, S. 2014 | ＊＊＊ | ＊＊ | ＊＊＊ | 8 |
| Gonzalez-Echavarri, C. 2015 | ＊＊＊ | ＊ | ＊＊ | 6 |
